# Phenotypic expressions of hereditary Transthyretin Ala97Ser related Amyloidosis (ATTR) in Taiwanese

**DOI:** 10.1186/s12883-017-0957-4

**Published:** 2017-09-07

**Authors:** Hui-Ching Hsu, Ming-Feng Liao, Jung-Lung Hsu, Ai-Lun Lo, Hung-Chou Kuo, Rong-Kuo Lyu, Victor Chien-Chia Wu, Chih-Wei Wang, Long-Sun Ro

**Affiliations:** 1 Department of Neurology, Chang Gung Memorial Hospital Linkou Medical Center and Chang Gung University College of Medicine, No.199, Tung Hwa N. Rd., Songshan Dist, Taipei City, 105 Taiwan, Republic of China; 2 Department of Traditional Chinese Medicine, Division of Chinese Acupuncture and Traumatology, Chang Gung Memorial Hospital Linkou Medical Center and Chang Gung University College of Medicine, No.5, Fuxing St., Guishan Dist, Taoyuan City, 333 Taiwan, Republic of China; 3 Department of Cardiology, Chang Gung Memorial Hospital Linkou Medical Center and Chang Gung University College of Medicine, No.5, Fuxing St., Guishan Dist, Taoyuan City, 333 Taiwan, Republic of China; 4 Department of Anatomic Pathology, Chang Gung Memorial Hospital Linkou Medical Center and Chang Gung University College of Medicine, No.5, Fuxing St., Guishan Dist, Taoyuan City, 333 Taiwan, Republic of China

**Keywords:** Familial amyloid polyneuropathy, Transthyretin-related amyloidosis, Ala97Ser Mutation

## Abstract

**Background:**

The disease course and early signs specific to ATTR Ala97Ser, the most common endemic mutation in Taiwan, have not been well described. Since new medications can slow down the rate of disease progression, the early diagnosis of this heterogeneous and fatal disease becomes critical.

**Methods:**

We retrospectively reviewed the characteristics of genetically confirmed ATTR Ala97Ser patients at a tertiary referral medical center.

**Results:**

Eight patients from 7 different families were enrolled (61.7 ± 5.5 years). Gastrointestinal symptoms, dyspnea or chest tightness, rather than sensory symptoms, were the initial symptoms in two patients (2/7 = 29%). Body weight loss (3/7 = 43%), muscle wasting (4/7 = 57%), or dysphagia (3/7 = 43%) were the consecutive symptoms. Orthostatic symptoms including orthostatic hypotension (7/7 = 100%), dizziness (6/7 = 86%) and syncope (5/7 = 71%) tended to develop in the late phase of the disease. Autonomic dysfunction was conspicuous. Cardiographic findings included a combination of ventricular wall thickening and pericardial effusion (7/7 = 100%), a granular sparkling appearance of the ventricular myocardium (4/7 = 57%), or conduction abnormalities (5/7 = 71%).

**Conclusions:**

This study broadens the recognition of the initial signs and symptoms, including cardiographic findings and longitudinal manifestations in Taiwanese individuals with ATTR Ala97Ser mutation. These manifestations should prompt doctors to perform further studies and make an early diagnosis.

**Electronic supplementary material:**

The online version of this article (10.1186/s12883-017-0957-4) contains supplementary material, which is available to authorized users.

## Background

Hereditary transthyretin-related amyloidosis (ATTR) is the most common form of familial systemic amyloidosis and is caused by dominantly inherited transthyretin (TTR) mutations [1–3]. ATTR is a rare but widely distributed disease with a clinical picture that varies in persons from different geographic, ethnic or genetic backgrounds, even among those with the same mutation or within the same family [4, 5]. Ala97Ser, the most common endemic mutation in Taiwan, is much less prevalent in the rest of the world. Only three articles (fewer than 60 cases in Taiwan) have been reported thus far [6–8]. The various clinical presentations of ATTR were available in the existing literature. Nevertheless, the early signs/symptoms specific to ATTR Ala97Ser and the progression of ATTR Ala97Ser over time have not been described previously.

Transthyretin mutations result in the deposition of amyloid fibrils in the peripheral and autonomic nerves, gastrointestinal tract and myocardium, typically presenting with an axonal predominant, length-dependent sensorimotor polyneuropathy (thus, the disorder is also termed familial amyloid polyneuropathy, FAP [2]) that frequently coexists with autonomic dysfunction, cardiac involvement and weight loss [9].

In addition, because emerging data suggest that the TTR-stabilizers can be more beneficial when treatment begins earlier [10, 11], there is an increasing necessity to identify the early signs and symptoms of this progressive and fatal disease so that early diagnosis can be made and treatment can be initiated earlier.

## Methods

### Clinical data

This research was carried out in accordance with the guidelines and regulations approved by the Institutional Review Board of Chang Gung Memorial Hospital and University (License no. 100-4470A3 and 104-2462A3). Informed consents were obtained from all patients. We enrolled patients with diagnoses of TTR Ala97Ser FAP based on a genetic analysis from 2010 to 2013 (see Additional file 1: Figure S1). We retrospectively reviewed the entire medical records in our hospital until October 2016 and compared clinical manifestations among subjects. We evaluated the onset age of the signs and symptoms and compared the sequential presentations among individuals. We reviewed other data, including the chief complaints, family history, clinics visited before the neurology referral, biopsy studies if available, reports of electrophysiological tests, electrocardiography (ECG), echocardiography findings, and major events including the placement of a permanent pacemaker and cardiopulmonary-cerebral resuscitation (CPCR). Positive ratios of the clinical data reported in two published articles of Ala97Ser in Taiwan were also compared with the results of the present study.

### Genetic analyses of mutations

Genomic DNA was isolated from the peripheral blood samples. The intronic primers applied to expand the promoter region, and all exons of the TTR gene were chosen according to previous research [12]. Polymerase chain reaction (PCR) used Tag ‘T’ DNA polymerase. Amplification proceeded for 29 cycles with denaturation at 94 °C for 30 s, annealing at 51 °C for 30 s, and extension at 72 °C for 45 s. There was a 10-min post-elongation at 72 °C. All PCR products were alkali denatured and submitted to electrophoresis in a 1× TBE buffer. Electrophoresis was performed at room temperature. The PCR products were directly sequenced by an autosequencing machine (DNA Engine, Platier Thermal Cycler, Hercules, CA, USA).

### Histopathology

Patients received colonoscopic or salivary gland biopsies if amyloidosis was suspected. The slides of hematoxylin and eosin stain were examined. Congo red staining was then performed and the slides were viewed under a polarized microscope light. (LEITZ LABORLUX S, Solmes, Germany).

## Results

### Clinical manifestations and progression of ATTR Ala97Ser

All patients gradually developed peripheral neuropathy, including progressive tingling, occasional throbbing/shooting pain, or paresthesia/dysesthesia in the feet. Symptoms started in the distal parts of the lower limbs and then progressed to the upper limbs, with resultant walking difficulty (5/7, 71%) and/or functional impairment of the hands (3/7, 43%) in variable time intervals (see Table 1).

**Table 1:**
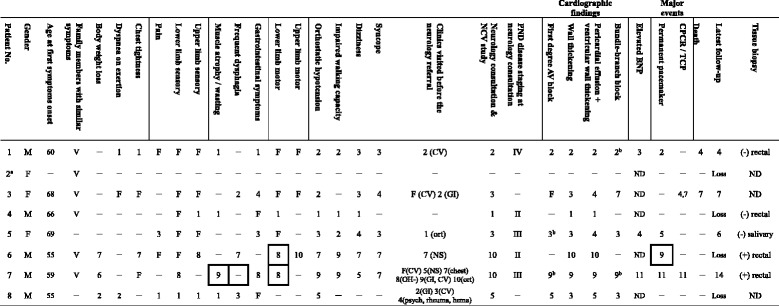
Clinical course of patients with ATTR Ala97Ser mutation

For the sake of simplicity, an “F” stands for the year of the first symptoms or findings for each subject, and the ordinal number in each row stands for the interval (years) between the onset of the first and consecutive symptoms or events. The progressions in individuals are illustrated in rows

^a^Patient 2 is the daughter of patient 1, and patient 2 was excluded from the age analysis because she had normal NCV study findings

^b^The patients had developed higher degree AV block or complete BBB during the observation period. Please see the Results section

CPCR/TCP: cardiopulmonary-cerebral resuscitation or transcutaneous cardiac pacing use

*CV* Cardiology, *GI* Gastrology, *ort* Orthopedics, *NS* Neurosurgeon, *chest* chest, OH-: negative results after survey at other hospital, psych: psychiatrist, rheuma: rheumatology, hema: hematology

Values that are enclosed indicate patient who reported similar symptoms/events in their family members

PND disease staging [5]: polyneuropathy disability staging, Stage 0: no impairment, Stage I: sensory disturbances but preserved walking capability, Stage II: impaired walking capability but ability to walk without a stick or crutches, Stage IIIA: walking only with the help of one stick or crutch, Stage IIIB: walking with the help of two sticks or crutches, Stage IV: confined to a wheelchair or bedridden

ND: not done

Gastrointestinal (GI) symptoms (6/7, 86%), dyspnea on exertion (3/7, 43%), or chest tightness (4/7, 57%), rather than sensory symptoms, were the initial symptoms in patients 7 and 8 (Table 1). GI symptoms were common and included chronic constipation, diarrhea or constipation alternating with diarrhea [5, 7]. Chest tightness occurred particularly at night (with orthopnea). Patient 7 complained of chest tightness over the substernal area with cold sweating for up to 2 h. Cardiac involvement was common and could lead to symptoms of heart failure. Patients 1, 5 and 7 presented with elevated brain natriuretic peptide (BNP) [5] at levels up to 200, 108, and 232 pg/ml at 3, 4, and 11 years after the initial symptom onset, respectively (Table 1).

The progression of the disease was accompanied by the occurrence of body weight loss (BWL), muscle wasting, or dysphagia. Persistent BWL of up to 20 kg were found within a duration of 3 ~ 20 years. Patients may have suffered from GI symptoms or unexplained BWL before typical neurological symptoms presented. Muscle atrophy/wasting (4/7, 57%) bilaterally affected the hands, thighs, or shoulder girdles. Frequent choking or progressive dysphagia were not related to stroke (normal brain MRIs). A negative panendoscope and PET scan results in patient 6 excluded the presence of an esophageal tumor. Esophagography revealed probable esophageal dysmotility in patient 3 [3]. Patient 7 did not develop dysphagia, but his brother did. Family members with similar symptoms were observed in six patients (6/8, 75%; Table 1).

Orthostatic hypotension, dizziness and recurrent syncope usually occurred in the late phase of the disease. Exertional syncope usually lasted for seconds and occurred during defecation, after voiding [13], after deep breathing, standing from a sitting position, or while driving. Cold sweat and decreased blood pressure (systolic blood pressure 70 ~ 80 mmHg) often accompanied frequent falls [13].

**Fig. 1:**
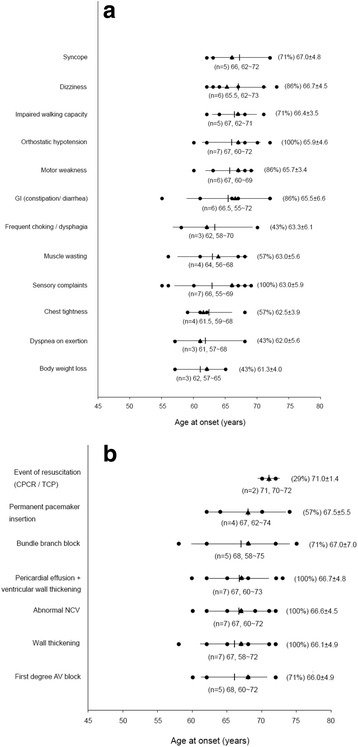
The onset age of warning symptoms (**a**) and signs (**b**) in patients with ATTR Ala97Ser mutation. Data are presented as (patient number), median, range below the bars and (%), mean ± standard deviation on the right side of the bars. Circles: age at onset of each subject; ▲symbols: median; bars with vertical line: mean ± standard deviation

The unfavorable events (syncope/cardiac arrest/CPCR) occurred approximately 4 years after the appearance of the initial neurological symptoms (Fig. 1). CPCR or temporary transcutaneous cardiac pacing (TCP) implantation were observed in three episodes. Patient 3 was implanted with a TCP device at our emergency department at the age of 72 because of a sudden sinus arrest (a slow junctional escape rhythm with shock status), which was suspected to result from an amiodarone-related side effect. Patient 7 was revived with CPCR after sudden cardiac arrest. Ventricular tachyarrhythmia and high degree atrioventricular (AV) block were detected in this patient.

Patients 5 and 7 were alive as the present study ended and returned to clinics for regular follow-up appointments. Patients 1 and 3 died from severe sepsis.

### Paraclinical and histopathological findings

#### Electrophysiological tests

The most common finding on nerve conduction studies was an axonal predominant, length-dependent sensorimotor polyneuropathy (7/7, 100%). Bilateral carpal tunnel syndromes were detected in patients 4, 6 and 8. In contrast, patient 2 had a normal nerve conduction study at the age of 37. Current perception threshold testing (CPT) of 4 patients revealed the dysfunction of both large and small sensory nerves in 3 patients (3/4, 75%) and the dysfunction of A beta-fibers of both feet in one patient (1/4, 25%). A sympathetic skin response (SSR) was absent in the palms and soles of five patients (5/6, 83%) and in the soles alone of one patient (1/6, 17%), among the 6 patients who were evaluated for SSR.

#### Histopathology

**Fig. 2:**
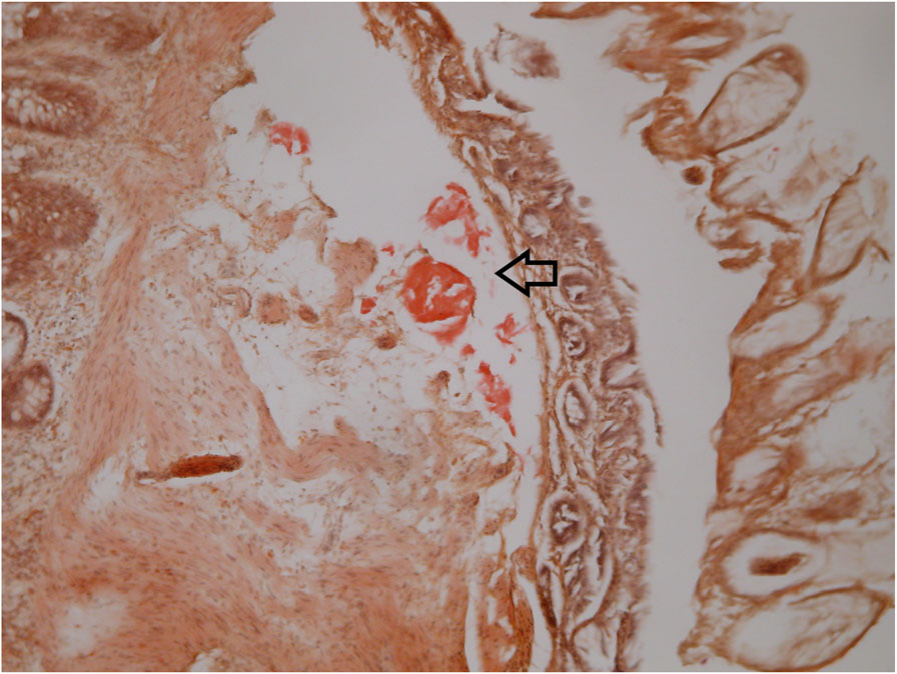
A representative colonic biopsy of patient 6 showed a portion of amyloid deposits in the submucosa; the deposits stained positive for Congo red stain (arrow, 100X)

Colonic tissue biopsies were positive for Congo red staining in patients 6 and 7 (Fig. 2). Amyloid fibrils were not found on Congo red stained sections of the other three patients.

#### Electrocardiography and echocardiography

**Fig. 3:**
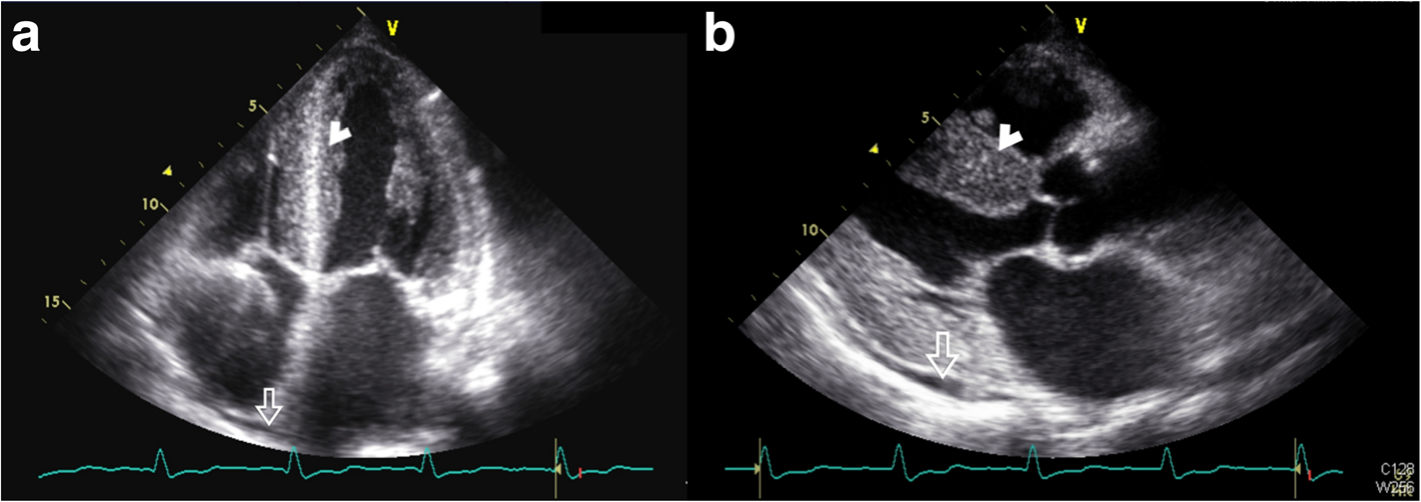
**a** In an apical 4-chamber view, biventricular wall thickening, granular sparkling (arrowhead), thickening of the mitral and tricuspid valves, and a trivial amount of pericardial effusion next to the right atrium are visible in the systolic phase (arrow) (patient 6, age 65). **b** In a parasternal long-axis view, a left atrium dilatation and a very thick granular myocardium (arrowhead) with some pericardial effusion (arrow) at the baso-posterior wall are evident (patient 6, age 65)

**Fig. 4:**
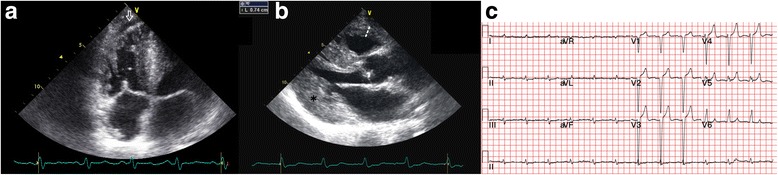
**a** In a right ventricle-centered view, a thickened right ventricular wall and a small amount of pericardial effusion next to the right ventricular apex (arrow) are apparent (patient 7, age 68). Among the serial echocardiographic studies this patient received, this is the earliest echo image in which the combination of ventricular wall thickening and pericardial effusion can be identified. **b** In this parasternal long-axis view, a thickening of the right ventricular free wall at 0.74 cm (dashed line) and very thick left ventricular walls (asterisk) are apparent (patient 7, age 69). **c** The ECG of this patient shows a normal sinus rhythm and a complete left bundle branch block (age 69)

In our study, the most common ECG and echocardiography findings, including pericardial effusion associated with progressive ventricular wall thickening (7/7, 100%; Fig. 3 and Fig. 4) [2], AV block (5/7, 71%), and bundle-branch block (BBB; 5/7, 71%; Table 1).

All patients exhibited progressive left ventricular (LV) wall thickening on echocardiography, but none had electrocardiographic signs of LV hypertrophy. Only two patients had low voltage QRS in the limb leads and the precordial leads, respectively [2]. Echocardiogram a granular sparkling appearance of the ventricular myocardium in four patients (57%; Fig. 3) [2, 4, 14]. Concentric LV hypertrophy was noted in patients 3, 4, 5 and 7 [2]. Except for patient 3, none had systemic hypertension that could explain ventricular hypertrophy [2].

Patients 1 and 7 developed complete left BBB. Patient 8 had left anterior fascicular block. Patients 3 and 5 had right BBB. Patients 5 and 7 developed second- and third-degree AV block at 3 and 11 years after the initial symptom onset, respectively [15]. Pan-conduction system disease was also identified in patient 1. Other findings included left atrial enlargement (patients 1, 4, 6 and 7; Fig. 3) [15], atrial fibrillation (patients 1 and 7) [2], and left axis deviation (patients 5 and 8) [15].

### Search for a constellation of progressive manifestations as warning symptoms

Table 1 lists common manifestations and warning symptoms and reveals a rapid progression of autonomic dysfunction and cardiac involvement along with progressive polyneuropathy. Core features of clinical and laboratory manifestations were summarized in Table 2.

**Table 2:** Core features of the clinical and laboratory manifestations of patients with ATTR Ala97Ser mutation

	(n = number of patients, males: females)	This study (*n* = 8, 5:3)	Liu et al., 2008 (*n* = 5, 3:2)	Chao et al., 2015 (*n* = 28, 25:3)
Core features	1. Late onset age (years, mean ± SD, median, range)	61.7 ± 5.5, 60, 55–69	50.4 ± 5.6,49, 46–60	59.9 ± 6.0,59, 48–71
	2. Progressive polyneuropathy^a^	7/7 (100.0%)	5/5 (100.0%)	28/28 (100.0%)
	3. Progressive autonomic dysfunction^b^	7/7 (100.0%)	5/5 (100.0%)	22/28 (78.6%)
	4. Family members with similar symptoms or documented ATTR	6/8 (75.0%)	5/5 (100.0%)	NA
Supportive laboratory findings	1. Progressive axonal predominant, length-dependent, sensorimotor polyneuropathy on electrophysiology	7/7 (100.0%)	5/5 (100.0%)	28/28 (100.0%)
	2. Ventricular wall thickening in association with pericardial effusion on echocardiography	7/7 (100.0%)	NA	NA
	3. LVH in association with low to normal QRS voltage on electrocardiography	7/7 (100.0%)	NA	NA
	4. Granular sparkling appearance of the ventricular myocardium on echocardiography	4/7 (57.1%)	NA	NA
	5. Hypertrophic cardiomyopathy in the absence of other causes^c^	7/7 (100.0%)	3/5 (60.0%)	NA
	6. Conduction abnormalities on ECG	5/7 (71.4%)	3/5 (60.0%)	NA
	7. Amyloid deposition on tissue biopsy	2/5 (40.0%)	3/5 (60.0%)	> 1/3 (> 33.3%)

^a^Data are presented as ratio. The denominators differ according to the availability of the data. NA: not available

^b^Autonomic dysfunction including gastrointestinal (constipation, diarrhea, or nausea/vomiting), orthostatic (hypotension, dizziness, syncope) and genitourinary symptoms (sexual dysfunction, urinary incontinence, urinary retention)

^c^Increased thickness of the ventricular wall, atrial septum, interventricular septum, or atrioventricular valve

## Discussion

In this study, we aimed to describe a constellation of easily recognized manifestations that could alert physicians to diagnose ATTR Ala97Ser in Taiwan. Patients initially presented with warning symptoms that might be easily overlooked (including GI symptoms, dyspnea, chest tightness, body weight loss, muscle wasting, or dysphagia). A rapid progressive course was conspicuous and included autonomic disturbances, severe axonal predominant, length-dependent sensorimotor polyneuropathy, and abnormal cardiographic findings [16]. The presence of these manifestations in patients 50–60 years old, as well as similar symptoms among family members, should prompt clinicians to consider the probability of a diagnosis of ATTR.

An intriguing observation was that most patients visited different clinics before receiving a final diagnosis at a neurology department of a referral hospital (Table 1). Cardiologists and gastrologists were most frequently consulted because of chief concerns, the warning symptoms, including chest tightness, dyspnea, dizziness, BWL, constipation (or diarrhea), or frequent choking. These findings revealed a serious issue: patients did not consult a neurologist for their sensory complaints until neurologic symptoms became apparent. Subspecialists did not connect the patients’ chief concerns with ATTR, and thus did not vigilantly investigate sensory deficits, family history or other manifestations listed in Table 1. However, the diagnosis of ATTR is particularly challenging in patients without overt neurological symptoms [2]. TTR-FAP can present as non-familial particularly in late-onset cases [1, 17].

Newly observed presentations or events that had not been reported regarding ATTR Ala97Ser in Taiwan include occasional shooting pain or tingling accompanied with lower limb numbness, dyspnea, chest tightness, frequent choking or dysphagia, dizziness, frequent episodes of syncope, abnormal cardiographic findings (Table 2), and CPCR. Other presentations or events documented in this study as well as by Liu’s study [7] are corroborated and worth our attention; these include muscle atrophy resulting from denervation [1, 18], BWL, permanent pacemaker placement, and a preceding carpal tunnel syndrome [5].

Syncope is predisposed by induction of a Valsalva maneuver and is a risk factor for sudden cardiac death [13, 19, 20]. The high ratio of autonomic dysfunction (Table 2) and the exacerbations of orthostatic symptoms suggest that autonomic dysfunction in ATTR Ala97Ser may not be as mild as other types of late-onset FAP [1, 7].

The serial ECGs and/or echocardiography that are often the initial work-ups in these patients play an important role in providing supportive clues since late-onset TTR-related amyloidosis often has cardiac involvement [16, 18, 21]. An ECG or echocardiography finding alone is relatively nonspecific, but the combination of clinical presentations can be more suggestive of ATTR Ala97Ser. Possible sampling errors, the uneven distribution of amyloid fibrils, and the predominant non-fibrillar amyloid precursor material in late-onset specimens may lead to a false negative finding and the low positive ratio of tissue biopsies in this study [5, 17, 18]. Therefore, DNA testing, the most reliable test for TTR-FAP, should be performed without being misled by a negative biopsy in patients with clinical suspicion of TTR-FAP [1, 17].

ATTR Ala97Ser appears to be a late-onset disorder [18, 22, 23]. Nonetheless, there are some differences between ATTR Ala97Ser and other amyloidoses. Left BBB was not as rare as other TTR variants [2]. Unlike light-chain (AL) amyloidosis and early-onset TTR Met 30 FAP [23], renal impairment was less frequent in our subjects. Unlike other TTR variants [1], ocular manifestations were also rare; only patient 5 had glaucoma [24].

There are some limitations in this study. First, the data were collected from a single referral medical center. Second, as this was a retrospective chart review, clinical information and measures were not uniform. Repeated neuropathy impairment score (NIS) and modified body mass index (mBMI) that can reflect disease progression were incomplete [25]. Laboratory studies were not performed early or serially. Three subjects refused biopsy due to personal reasons. No one received a postmortem examination. According to recent research, bone scintigraphy for detection of ATTR cardiac amyloidosis should be performed in the future, because it may replace the traditional requirement for histological confirmation [26]. Third, we were unable to collect complete longitudinal data on patients who were followed up at other hospitals because of personal preference or convenience.

## Conclusion

Only a limited number of ATTR Ala97Ser patients have been reported to date in Taiwan. Although rare, careful clinical characterization can facilitate early diagnosis and intervention, which can improve the prognosis of patients with ATTR Ala97Ser. Thus, a further nationwide, prospective enrollment and data collection is warranted.

## Additional file

Additional file 1: Figure S1. The common (missense) mutation Ala97Ser was demonstrated by directly sequencing exon 4 of the human TTR gene. The arrow indicates DNA nucleotide T substitution for G. (PDF 108 kb)

## Abbreviations

ATTR: Hereditary transthyretin related amyloidosis
AV: Atrioventricular
BBB: Bundle-branch block
BNP: brain natriuretic peptide
BWL: Body weight loss
CPCR: Cardiopulmonary-cerebral resuscitation
CPT: Current perception threshold testing
ECG: Electrocardiography
FAP: Familial amyloid polyneuropathy
GI: Gastrointestinal
LV: Left ventricular
mBMI: Modified body mass index
NIS: Neuropathy impairment score
SSR: Sympathetic skin response
TCP: Temporary transcutaneous cardiac pacing
TTR: Transthyretin
