# Supplementary material for: Phenotypic expressions of hereditary Transthyretin Ala97Ser related Amyloidosis (ATTR) in Taiwanese
Source: BMC Neurol. 2017 Sep 7;17:178. doi: 10.1186/s12883-017-0957-4 (PMC5590125; doi:10.1186/s12883-017-0957-4)

Additional file 1: Figure S1.

The common (missense) mutation Ala97Ser was demonstrated by directly sequencing exon 4 of the human TTR gene. The arrow indicates DNA nucleotide T substitution for G.

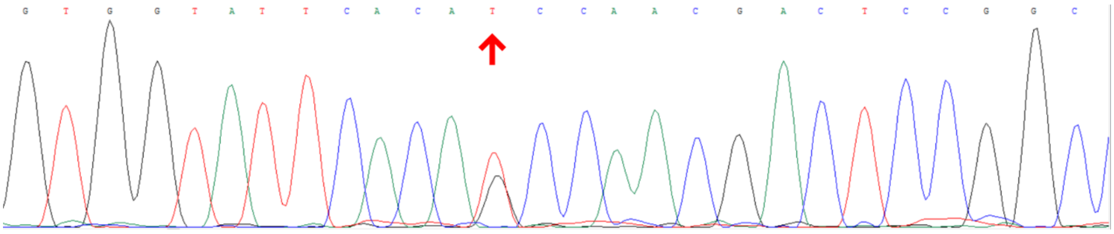

Supplement: Additional file 1: Figure S1. — The common (missense) mutation Ala97Ser was demonstrated by directly sequencing exon 4 of the human TTR gene. The arrow indicates DNA nucleotide T substitution for G. (PDF 108 kb) [file 12883_2017_957_MOESM1_ESM.pdf]
